# The involuntary nature of binge drinking: goal directedness and awareness of intention

**DOI:** 10.1111/adb.12505

**Published:** 2017-04-16

**Authors:** Nuria Doñamayor, Daniela Strelchuk, Kwangyeol Baek, Paula Banca, Valerie Voon

**Affiliations:** ^1^ Department of Psychiatry University of Cambridge Cambridge UK; ^2^ Department of Biomedical Engineering Ulsan National Institute of Science and Technology Ulsan Korea; ^3^ Behavioural and Clinical Neurosciences Institute Cambridge UK; ^4^ Cambridgeshire and Peterborough NHS Foundation Trust Cambridge UK; ^5^ NIHR Cambridge Biomedical Research Centre Cambridge UK

**Keywords:** alcohol dependence, binge drinking, goal‐directed behaviour, habitual behaviour, intention awareness

## Abstract

Binge drinking represents a public health issue and is a known risk factor in the development of alcohol use disorders. Previous studies have shown behavioural as well as neuroanatomical alterations associated with binge drinking. Here, we address the question of the automaticity or involuntary nature of the behaviour by assessing goal‐directed behaviour and intentionality. In this study, we used a computational two‐step task, designed to discern between model‐based/goal‐directed and model‐free/habitual behaviours, and the classic Libet clock task, to study intention awareness, in a sample of 31 severe binge drinkers (BD) and 35 matched healthy volunteers. We observed that BD had impaired goal‐directed behaviour in the two‐step task compared with healthy volunteers. In the Libet clock task, BD showed delayed intention awareness. Further, we demonstrated that alcohol use severity, as reflected by the alcohol use disorders identification test, correlated with decreased conscious awareness of volitional intention in BD, although it was unrelated to performance on the two‐step task. However, the time elapsed since the last drinking binge influenced the model‐free scores, with BD showing less habitual behaviour after longer abstinence. Our findings suggest that the implementation of goal‐directed strategies and the awareness of volitional intention are affected in current heavy alcohol users. However, the modulation of these impairments by alcohol use severity and abstinence suggests a state effect of alcohol use in these measures and that top‐down volitional control might be ameliorated with alcohol use cessation.

## Introduction

Binge drinking is characterized by consuming large quantities of alcohol (blood alcohol concentration ≥ 80 mg%) in a short period of time (about 2 hours) followed by longer periods of abstinence. It has numerous negative social and individual consequences and is a prominent risk factor for the development of alcohol abuse disorders (Crabbe, Harris & Koob 2011). Binge drinking has been associated with attentional impairments, decreased sensitivity to the anticipation of high‐risk negative outcomes and increased premature responding and trait impulsivity (Bauer & Ceballos 2014; Sanchez‐Roige *et al.* 2014; Worbe *et al.* 2014; Morris, Kundu, Baek, *et al.* 2016a). Neuroimaging has further revealed grey matter abnormalities within ventral striatum (VS), prefrontal cortex (PFC) and limbic areas in binge drinkers (BD) (Howell *et al.* 2013; Doallo *et al.* 2014; Mashhoon *et al.* 2014), as well as PFC hyperactivity following high‐risk decisions (Worbe *et al.* 2014), but hypoactivity during working memory tasks (Crego *et al.* 2010). Several lines of evidence suggest potential abnormalities in involuntary automatic behaviours in addictions, from a shift away from goal‐directed behaviours and relapse triggers from incentive cues. Here, we assess the relationship between goal‐directed behaviours and motor intention in severe BD.

Dual‐system accounts suggest that action control is determined by the balance between goal‐directed or model‐based behaviour, driven by response–outcome information, and habitual or model‐free behaviour, driven by stimulus–response associations (Gläscher *et al.* 2010; van der Meer & Redish 2010; de Wit *et al.* 2012; Dolan & Dayan 2013). Overreliance on habits over model‐based behaviour has been reported in a number of neuropsychiatric disorders, including chronic addiction (Gillan *et al.* 2011; Hogarth, Chase & Baess 2012; Sjoerds *et al.* 2013; Gillan *et al.* 2014; Voon, Derbyshire, *et al.* 2015b). Furthermore, anatomical, functional and connectivity studies have evidenced an overlap between the neural substrate of the model‐based and model‐free systems, which involves lateral and ventromedial PFC, orbitofrontal cortex, inferior parietal cortex (IPC) and striatum (Valentin, Dickinson & O'Doherty 2007; Tanaka, Balleine & O'Doherty 2008; de Wit *et al.* 2009; Gläscher *et al.* 2010; de Wit *et al.* 2012; Voon, Derbyshire, *et al.* 2015b; Morris, Kundu, Dowell, *et al.* 2016b), and the structures altered in BD (Howell *et al.* 2013; Vargas *et al.* 2014; Whelan *et al.* 2014; Worbe *et al.* 2014; Morris, Kundu, Baek, *et al.* 2016a).

It has been shown that alcohol dependence affects the ability to base behaviour on response–outcome information (Sjoerds *et al.* 2013), which is at the core of model‐based behaviour. A further study showed that heavy drinkers, including alcohol‐dependent subjects, showed increased alcohol cue reactivity in the dorsal striatum, whereas light social drinkers showed increased VS and PFC activities (Vollstädt‐Klein *et al.* 2010), suggesting a shift from ventral to dorsal striatal involvement, such as that seen in the development of addiction, and from goal‐directedness to habitual behaviour (Corbit, Nie & Janak 2012; Everitt & Robbins 2013). Similarly, alcohol‐dependent subjects tested early in abstinence showed impaired model‐based behaviours tested using the two‐step task (Sebold *et al.* 2014). However, this bias towards model‐free behaviour in alcohol‐dependent individuals has been reported to decrease with prolonged abstinence, suggesting a possible role for top‐down volitional control in increasing goal directedness (Voon, Derbyshire, *et al.* 2015b). We have also previously shown that BD take more risks when anticipating loss outcomes. The behaviour shifts towards a more risk‐averse attitude when exposed explicitly to the actual loss outcome, suggesting potentially that the explicit nature of experiencing the loss might counteract underlying automatic biases towards risk (Worbe *et al.* 2014).

Model‐based behaviour involves active deliberation and prospective planning in order to direct actions towards desirable outcomes (Gläscher *et al.* 2010; van der Meer & Redish 2010; de Wit *et al.* 2012; Dolan & Dayan 2013). Therefore, the fact that individuals drink excessively or go in drinking binges, despite their negative consequences (Crabbe *et al.* 2011), appears to contradict successful goal‐directed behaviour. Furthermore, this ability to prospectively track motivationally relevant outcomes most probably also requires a certain level of volitional awareness, more specifically, of both the planning or intention to act and the action itself.

Intention awareness has been extensively studied in the field of motor intention. Libet *et al.* (1983) designed a task aimed at discerning between the awareness of the intention to move and of the movement itself, showing that the former (W judgement) occurred around 200 milliseconds before the latter (M judgement). Although their task has been subject to some criticism (Gomes 1998; Danquah, Farrell & O'Boyle 2008), numerous studies have replicated the basic findings of the original study (Lau *et al.* 2004; Walsh *et al.* 2010; Fried, Mukamel & Kreiman 2011). Anatomically, intention awareness has been reported to involve supplementary motor complex, lateral PFC and IPC (Fried *et al.* 1991; Lau *et al.* 2004; Fried *et al.* 2011; Desmurget & Sirigu 2012), thus sharing part of the neural substrate of model‐based behaviour.

The interval between W and M judgements can be used as an implicit measure of conscious awareness of volitional intention, and it has been argued that this interval would allow for intentional stopping or vetoing of the movement (Libet 1999; Haggard & Libet 2001). Several neuropsychiatric disorders have been associated with delayed awareness of motor intention (Edwards *et al.* 2011; Moretto *et al.* 2011; Tabu *et al.* 2015; Baek *et al.* in press), reflected as a reduced veto period or a shift of the W towards the M judgement. Moreover, this veto period might be used not only to stop the initiated movement but also to evaluate whether the selected action might be optimal to obtain the desired effect (Haggard & Libet 2001). As stated previously, optimal action selection is paramount to goal‐directed behaviour. Interestingly, at least two of the disorders associated with reduced W–M intervals, Parkinson's disease and Tourette's syndrome, have also been associated with alterations in model‐based and model‐free behaviours (Marsh *et al.* 2004; de Wit *et al.* 2011; Hadj‐Bouziane *et al.* 2013; Wylie *et al.* 2013), further supporting the notion that maintaining optimal balance between these two systems might require a certain level of intention awareness.

In the current study, we aimed to study goal‐directed behaviour and intention awareness in BD and healthy volunteers (HV). To this end, we used a two‐step task (Daw *et al.* 2011), specifically designed to computationally differentiate model‐free and model‐based behaviours, as well as the Libet clock task (Libet *et al.* 1983). We hypothesized that BD subjects with current ongoing heavy alcohol use would have lower goal‐directed learning compared to HV. We further hypothesized that BD would be impaired in intention awareness relative to HV.

## Materials and Methods

### Participants

Thirty‐one BD (12 women, 21.29 ± 2.52 years old) and 35 HV (20 women, 23.17 ± 2.83 years old) aged between 19 and 29 years were recruited from community and university‐based advertisements. Both groups were gender matched, χ^2^ *=* 2.24, *P >* 0.1, and age matched within 5 years, but the healthy controls were slightly older as a group, *t*_*y*_(51) = −3.22, *d =* 0.70, *P =* 0.002.

Binge‐drinking criteria were based on the National Institute on Alcohol Abuse and Alcoholism diagnostics that define consumption of greater than eight units for men or six units for women within a 2‐hour period at least once a week for the previous 6 months (National Institute on Alcohol Abuse and Alcoholism 2004, Winter). Subjects also had to have the intention to get drunk and also have been drunk at least once per week for the previous 6 months. They were excluded if they had a major psychiatric disorder, substance addiction or medical illness or were on psychotropic medications. Psychiatric disorders were screened with the mini‐international neuropsychiatric interview (Sheehan *et al.* 1998), and participants completed the National Adult Reading Test to assess verbal IQ (BD: *M* = 116.69, *SD* = 4.03, *n* = 26; HV: *M* = 115.47, *SD* = 5.66, *n* = 32; effect of group: *P >* 0.1; effect of the age covariate: *P >* 0.1), the Beck Depression Inventory to assess depressive symptoms (BD: *M* = 9.54, *SD* = 8.01, *n* = 26; HV: *M* = 7.29, *SD* = 7.48, *n* = 34; effect of group: *P >* 0.1; effect of the age covariate: *P >* 0.1) and the Alcohol Use Disorders Identification Test (AUDIT) (BD: *M* = 16.08, *SD* = 5.32, *n* = 26; HV: *M* = 4.53, *SD* = 3.34, *n* = 34; effect of group: *F*(1) = 86.33, *P <* 0.001; effect of the age covariate: *P >* 0.1) and the Alcohol Use Questionnaire to assess alcohol use severity (BD: *M* = 33.41, *SD* = 14.25, *n* = 22; HV: *M* = 8.87, *SD* = 8.90, *n* = 27; effect of group: *F*(1) = 57.35, *P <* 0.001; effect of the age covariate: *P >* 0.1). The mean number of times drunk over the last 6 months was greater for BD (BD: *M* = 20.70, *SD* = 14.25, *n* = 23; HV: *M* = 2.21, *SD* = 4.21, *n* = 34; effect of group: *F*(1) = 63.29, *P <* 0.001; effect of the age covariate: *P >* 0.1). Subjects were questioned on their patterns of alcohol consumption, to ensure they fit the criteria, and their last alcohol binge prior to testing was recorded. Subjects were administered a breathalyser test on the day of the study.

Participants were tested on two separate days, with the two‐step task tested on day 1 and the Libet clock and working memory tasks tested on day 2. All subjects provided written informed consent before enrolling and were compensated for their time. The study was approved by the University of Cambridge Research Ethics Committee.

### Task description

#### Two‐step task

**Figure 1:**
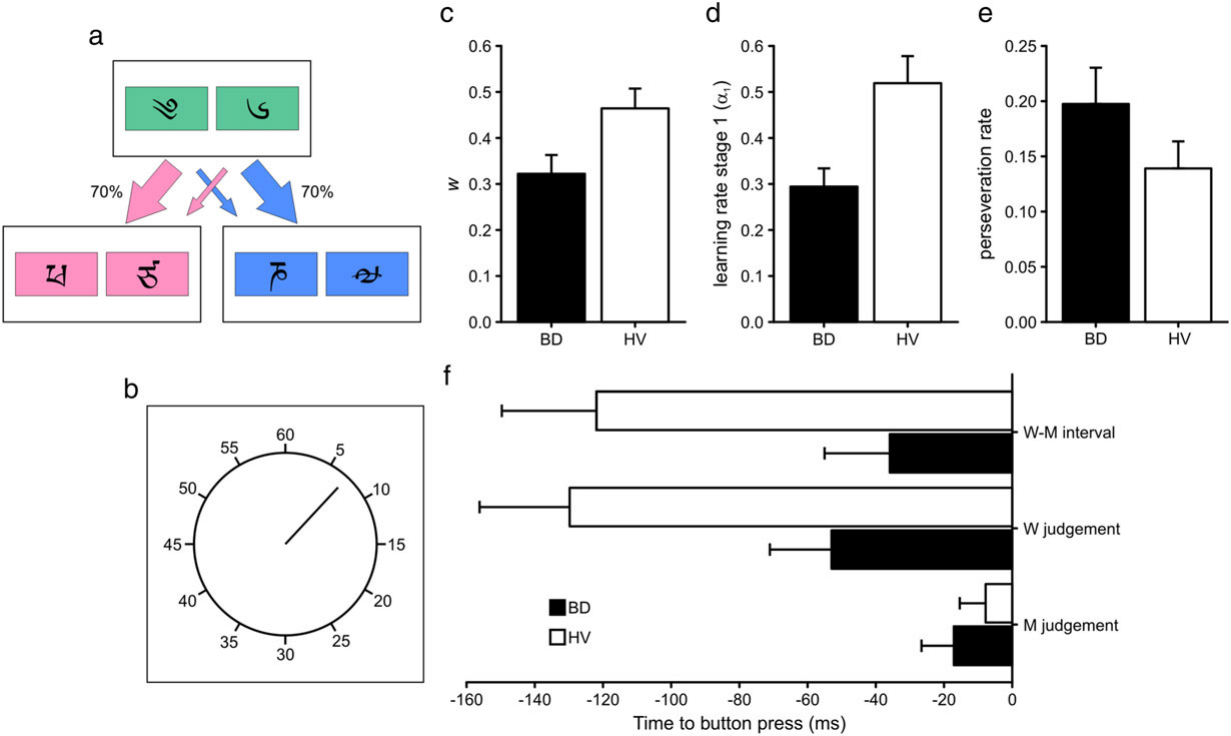
Results from the analysis of the two‐step and Libet clock tasks. *Schematic* depiction of the (a) two‐step and (b) Libet clock tasks. Mean (c) weighting parameter (*w*), (d) learning rate in stage 1 (α_1_) and (e) perseveration rate from the two‐step task. (f) Mean estimated times of intention (W judgement), movement (M judgement) and difference between intention and movement (W–M) relative to the recorded button press from the Libet clock task. Black bars represent binge drinkers (BD) and white healthy volunteers (HV). Error bars represent the standard error of the mean

Participants performed the two‐step task as described in our previous work (Fig. 1a) (Voon, Derbyshire, *et al.* 2015b). In stage 1, participants had to choose between two stimuli, each of which led to one of the two stimulus pairs with a fixed probability (*P =* 0.70) and to the other stimulus pair with opposite probability (*P =* 0.30). The stimulus choice made in stage 2 led to a reward with probability varying slowly and independently over time between 0.25 and 0.75. Each of the four stimuli in stage 2 was attached to a different probability distribution, and the association between each stage 2 stimulus and its reward probability was counterbalanced across participants. Choices at each stage had to be made within 2 seconds, and the result of each choice was presented after 1.5 seconds. The stimulus chosen in stages 1 and 2 remained on screen as a reminder in stage 2 and the outcome stage, respectively. If the stage 2 choice was rewarded, participants saw a £1 coin for 1 second; otherwise, they saw a grey circle for 1 second.

The task consisted of three blocks of 67 trials. Prior to the task, participants underwent extensive computer‐based instructions, which included explanatory examples of changes in transition and probability, and a short block of 50 trials in the same format as the experimental task but with different stimuli. The task was run with Cogent 2000 (http://www.vislab.ucl.ac.uk/cogent.php) on Matlab R2011a (MathWorks, Natick, USA).

#### Libet's clock task

In a separate session, participants also performed the Libet clock task (Fig. 1b) (Libet *et al.* 1983). In each trial, a hand revolved inside a clock face marked at 5‐minute intervals at 2560 milliseconds per cycle. Participants were instructed to make a button press with their right index finger after a random time interval. They were asked to act as spontaneously as possible and to avoid preselecting a position of the hand to trigger the button press.

Participants were required to make either a W judgement or an M judgement. In W judgement trials, they were instructed to pay attention to when they first felt the urge to press the button; in M judgement trials, they were instructed to pay attention to when they actually pressed the button. After the button press, the clock hand continued rotating for 1500–2500 milliseconds. Participants then verbally reported its position either where they first felt the urge to press the button or when they actually pressed it, depending on the type of trial.

The task consisted of one M judgement block followed by a W judgement block, each consisting of 20 trials. The programme was developed with LabView (National Instruments, Austin, TX, USA).

#### Memory tests

In order to control for possible working memory effects in the two‐step task, we also tested participants with the spatial working memory (SWM) and paired associates learning (PAL) tasks of the Cambridge Neuropsychological Test Automated Battery (http://www.cambridgecognition.com/technology).

### Statistical analysis

For the two‐step task, choice behaviour was fit to a computational model, which learned action values through both model‐based reinforcement learning and model‐free State‐Action‐Reward‐State‐Action (SARSA) (λ) temporal difference learning. Participants' choices were driven by the weighted combination of these two types of learning, with the relative weighting controlled by a free parameter *w*, which was used as a measure of the reliance on model‐free (*w* = 0) or model‐based (*w* = 1) strategies. Additional behavioural measures were the indices of choice reliability in each stage (β), the learning rate in each stage (α), the reinforcement eligibility parameter (λ) and the perseveration rate (for further information on the model and the equations used, see Daw *et al.* 2011; Voon, Baek, *et al.* 2015a). We also examined the behavioural parameters of model‐based (interaction between transition and outcome) and model‐free (effect of outcome) measures. Each parameter was analysed by means of analysis of covariance (ANCOVA) to test for differences between BD and HV, while controlling for the effect of the age covariate.

For Libet's clock task, average latencies (excluding values over/under 3 *SD*) were computed for each participant's W and M judgements. Latencies were introduced in a 2 × 2 model to test for the effects of group and type of judgement and using age as a covariate. The difference between W and M judgements (W–M interval) was used as an implicit measure of conscious awareness of volitional intention. This measure was tested using an ANCOVA with age as a covariate.

The relationship between test outcomes (*w*, model‐free and model‐based scores from the two‐step task, and W–M interval from the Libet clock task) and alcohol use severity, as reflected by AUDIT score, was subsequently tested on an exploratory basis. To this end, variables were introduced in regression models. If AUDIT score significantly predicted the behavioural measure, the Fisher *r*‐to‐*z* transformation was used to assess the significance of the difference between the correlation coefficients of BD and HV. Further, *k*‐means clustering was performed to test whether the days since the last binge drinking episode had an influence on the behavioural measures in the BD group. This method was chosen over regression because of the limited number of data points in the time variable.

Task outcomes were analysed using r (https://www.r‐project.org/). Data that did not conform to normality of distribution and/or had outliers were tested using robust statistical methods (Yuen's *t*‐test with 10 percent winsorization, robust rank‐based ANCOVA and least trimmed squares regression). Otherwise, parametric testing was used (linear regression). *k*‐means clustering used the Calinski–Harabasz index to estimate the number of clusters, with a maximum of 10 iterations and two clusters, given the reduced number of data points; the Duda–Hart test with a significance level of *P =* 0.005 was applied to assess whether the data set should be split into clusters.

## Results

### Two‐step task

Thirty BD and 35 HV completed the two‐step task. One BD did not complete this task.

After controlling for the effect of age, BD (*M* = 0.322, *SD* = 0.224) had significantly lower *w* scores than HV (*M* = 0.464, *SD* = 0.255), *F*(1) = 5.03, *P =* 0.029 (Fig. 1c); the age covariate was not significantly related to the *w* score, *P >* 0.1. However, when analysing the model‐based and model‐free scores separately, measures that are less robust than the computational analysis, there were no differences between groups in either score, *P >* 0.05, nor was the age covariate related to the scores, *P >* 0.05.

There were no significant differences in choice reliability in stage 1 (β_1_) between BD (*M* = 4.63, *SD* = 2.97) and HV (*M* = 5.69, *SD* = 3.78), *P >* 0.05, but there was a significant relationship between this parameter and the age covariate, *F*(1) = 6.43, *P =* 0.014. There were no differences in choice reliability in stage 2 (β_2_), nor was this parameter related to the age covariate, *P >* 0.1.

After controlling for the effect of age, BD (*M* = 0.294, *SD* = 0.217) also had significantly lower learning rates in stage 1 (α_1_) than HV (*M* = 0.519, *SD* = 0.348), *F*(1) = 4.68, *P =* 0.034 (Fig. 1d); but there was no relationship between α_1_ and participants' age, *P >* 0.1. There were no differences between the groups' learning rates in stage 2 (α_2_), nor was this parameter related to the age covariate, *P >* 0.1.

Binge drinkers (*M* = 0.197, *SD* = 0.180) had significantly higher perseveration rates than HV (*M* = 0.139, *SD* = 0.145) after controlling for the effect of age, *F*(1) = 4.21, *P =* 0.044 (Fig. 1e); but age was not related to perseveration rates, *P >* 0.1.

There were no significant differences between groups in the λ parameter, nor was this parameter related to the subjects' age, *P >* 0.1.

### Libet's clock task

Twenty‐two BD and 31 HV completed the Libet clock task. Nine BD and four HV did not complete this task, which was tested on a separate day.

The robust ANCOVA performed on the latencies of the W and M judgements showed the main effects of the group, *F*(1) = 5.40, *P =* 0.022, and condition, *F*(1) = 18.31, *P <* 0.001, and an interaction between the group and condition, *F*(1)*=*5.73, *P =* 0.018, after controlling for the effect of age. The age covariate had no relationship with the latencies of the W and M judgements, *P >* 0.05. Šidák‐corrected post hoc *t*‐tests showed that BD (*M* = −53 milliseconds, *SD* = 85) made the W judgement marginally closer to the actual movement than HV (*M* = −130 milliseconds, *SD* = 147), *t*_*y*_(41) = 2.15, *d* = 0.61, *P =* 0.074, whereas there were no differences in the latencies of the M judgement (BD: *M* = −17, *SD* = 45; HV: *M* = −8, *SD* = 42), *P >* 0.1.

After controlling for the effect of age, the critical difference between W and M judgements (W–M interval) was marginally shorter in the case of BD (*M* = −36 milliseconds, *SD* = 90) than in the case of HV (*M* = −122 milliseconds, *SD* = 155), *F*(1) = 4.00, *P =* 0.051, while the age covariate was not significantly related to the W–M interval, *P >* 0.1 (Fig. 1f).

### Memory

Twenty‐one and 22 BD completed the SWM and PAL tasks, respectively, and 23 HV completed both tasks.

After controlling for the effect of age, there were no statistical differences between the number of errors committed by BD and HV in the SWM task, *P >* 0.1, but the age covariate was significantly related to the number of errors, *F*(1) = 6.53, *P =* 0.014. There were neither differences between the number of errors committed by the groups in the PAL task nor an effect of the age covariate, *P >* 0.05.

### Relationship with clinical variables

**Figure 2:**
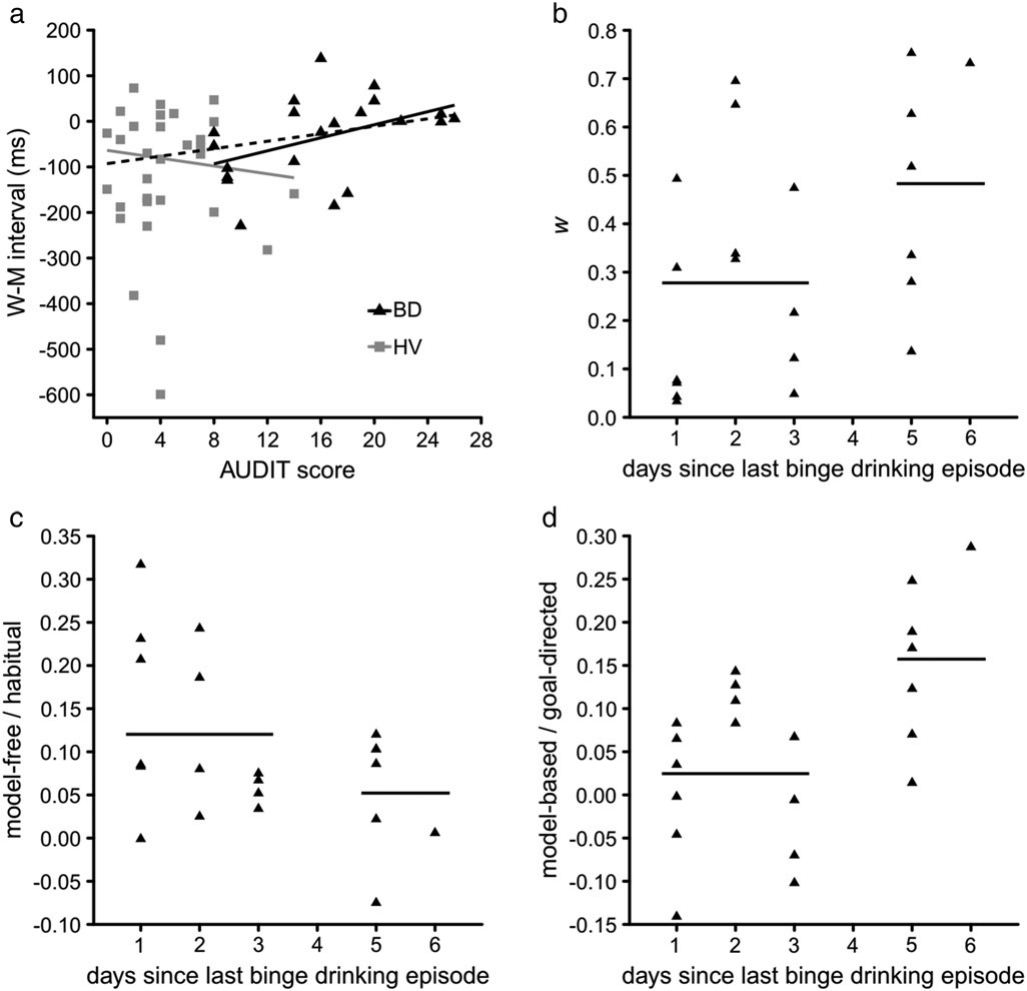
Results of the regression and cluster analyses. (a) Relationship between AUDIT score and W–M difference. Results of the cluster analysis of the (b) weighting parameter (*w*), (c) model‐free and (d) model‐based scores based on the days since the last binge drinking episode of the binge drinkers (BD). Horizontal lines represent the mean score of each cluster. BD are represented by black triangles and solid lines, healthy volunteers (HV) are represented by grey squares and solid lines and the regression line from the analysis over all subjects is represented by a black dashed line

On an exploratory basis, we further examined the relationship between the main outcomes and clinical variables. Regarding the Libet clock task, AUDIT score significantly predicted the length of the W–M interval (Fig. 2a), *F*(1, 47) = 4.96, *r*_*lts*_ = 0.31, *P =* 0.031, with greater alcohol use severity predicting shorter to inverted W–M intervals. AUDIT score significantly predicted the length of the W–M interval in the BD group, *F*(1, 19) = 4.82, *r*_*lm*_ = 0.45, *P =* 0.041, with greater alcohol use severity predicting shorter to inverted W–M intervals. However, AUDIT score was uncorrelated to W–M length in HV, *F*(1, 26) = 0.63, *r*_*lts*_ = −0.15, *P >* 0.1. The difference between the correlation coefficients of both groups was also statistically significant, *z* = 2.14, *P =* 0.032.

There was no relationship between AUDIT and either *w*, model‐free or model‐based scores from the two‐step task, *P >* 0.1.

Twenty‐two BD responded to the question regarding the days elapsed since their last binge. *k*‐means clustering showed that subjects that had had the last binge drinking episode under 4 days prior to testing had lower *w* (*M* = 0.278, *SD* = 0.229; Fig. 2b), as well as higher model‐free (*M* = 0.120, *SD* = 0.097; Fig. 2c) and lower model‐based (*M* = 0.025, *SD* = 0.088; Fig. 2d) scores than those that had had the last episode over 4 days before testing (*w*: *M* = 0.483, *SD* = 0.238; model‐free: *M* = 0.052, *SD* = 0.071; model‐based: *M* = 0.157, *SD* = 0.096), *P =* 0.002, *P =* 0.001 and *P =* 0.001, respectively.

The time since the last drinking binge had no influence on the W–M interval from Libet's clock task.

## Discussion

In the current study, we explore model‐based behaviour and intention awareness in BD and HV. Our BD sample shows particularly severe binge drinking behaviour, as they were only included in the study if they fit National Institute on Alcohol Abuse and Alcoholism criteria and further had been drunk at least once per week for the previous 6 months. The two‐step task (Daw *et al.* 2011) is used to differentiate between model‐based goal‐directed behaviour and model‐free habitual behaviour. In this task, BD show significantly lower *w* scores than HV, indicating a bias away from model‐based behaviours towards habitual behaviour. BD also showed lower learning rates than HV in stage 1 of the task, but not in stage 2. Once age was controlled for, we further show that BD have greater perseverative deficits, suggesting not only a tendency towards selecting the same previously rewarded action but also a greater tendency towards selecting the same stage 1 option irrespective of the outcome. The Libet clock task (Libet *et al.* 1983) is used to discriminate intention from movement awareness. Our findings indicate that, whereas M judgements are comparable between groups, the latency of the W or intention judgement in BD is shifted towards movement onset. Similarly, the W–M interval, an implicit measure of conscious awareness of volitional intention, is significantly reduced in BD than in HV. Crucially, we show that clinical variables appear to influence these measures in BD, with greater alcohol use severity predicting decreased conscious awareness of volitional intention and model‐freeness decreasing with the number of days after a binge episode.

A similar tendency towards habitual learning to the one observed here in BD has been previously observed in patients with obsessive–compulsive disorder, methamphetamine addicts and subjects with binge eating disorder (Voon, Derbyshire, *et al.* 2015b). However, studies with alcohol‐dependent individuals have offered inconsistent results. Sjoerds *et al.* (2013) reported impaired goal‐directed learning in alcohol‐dependent subjects using an instrumental learning task. Following overtraining on stimulus–response–outcome contingencies, subjects underwent an outcome‐devaluation test. The impairment in goal‐directed learning in alcohol‐dependent subjects was associated with decreased activity in the ventromedial PFC and anterior putamen, and increased activity in posterior putamen (Sjoerds *et al.* 2013). Using the two‐step task, Sebold *et al.* (2014) showed impaired model‐based behaviours in alcohol‐dependent subjects tested early in abstinence (2 weeks). However, Voon, Derbyshire, *et al.* (2015b) did not find differences between alcohol‐dependent subjects and HV, which may be related to the timing of testing, as they showed that *w* scores increased with abstinence duration (2 weeks to 1 year), suggesting an improvement in goal‐directed behaviour with abstinence. In the current study, we observe that those BD who had been abstinent for longer had higher *w* and model‐based scores, and lower model‐free scores than the rest of the group. Together, these findings suggest that individuals with current heavy alcohol use and binge drinking behaviours rely less on goal‐directed behaviours. The fact that abstinence and the number of days after a binge episode improve these scores points to a state effect of alcohol use on this measure.

Our BD sample further displayed significantly reduced W–M intervals compared with HV, suggesting an altered experience of volition. Similar results have been described in disorders of the basal ganglia (Moretto *et al.* 2011; Tabu *et al.* 2015), stroke lesions of the IPC (Sirigu *et al.* 2004) and functional neurological disorder (Edwards *et al.* 2011; Baek *et al.* in press), but to our knowledge, intention awareness had not yet been explored in relation to alcohol use disorders. Furthermore, our data show that alcohol use severity in BD is associated with poorer intention awareness.

There are several plausible mechanisms by which both of these processes may be impaired. We show that working memory is unlikely to be playing a role. Similarly, non‐specific attentional deficits would be likely to influence attention to both W and M measures, whereas these findings are specific to a shift in W in the BD group. Impairments in inhibitory control are common in substance use disorders and may be relevant across both tasks. The relevance of the interval between the awareness of intention and movement has been highlighted in the veto hypothesis (Libet 1999; Haggard & Libet 2001), which suggests that the 200 milliseconds interval between W and M can be used to evaluate and change or stop the initiated movement. Therefore, the W–M interval allows for the implementation of cognitive control mechanisms, which are required to avoid impulsive or compulsive acts (Bari & Robbins 2013) and execute goal‐directed behaviour. These findings also build on studies focusing on impaired awareness in substance use disorders on multiple levels, including awareness of emotional states, autonomic and bodily signals, error monitoring, awareness of deficits and behavioural choices, and higher‐order metacognitive judgements of confidence. Methamphetamine‐dependent subjects show elevated alexithymia or impaired emotional awareness, with an abnormal relationship with dopamine receptor availability in the anterior insula and anterior cingulate relative to healthy controls (Okita *et al.* 2016). The role of the anterior insula, implicated in conscious awareness of bodily and autonomic signals, is relevant to addictions (Verdejo‐García, Clark & Dunn 2012; Paulus & Stewart 2014; Stewart *et al.* 2014) and has been implicated in stroke lesion studies (Naqvi *et al.* 2007), hypoactivity during cognitive control and negative emotions, and decreased anterior insular volumes (Senatorov *et al.* 2015). Impairments in self‐monitoring of error awareness have been related to hypoactivity in the anterior cingulate and insula (Hester, Nestor & Garavan 2009). Impairments in higher‐order metacognitive self‐awareness were shown with cocaine use disorder with impairments in the relationship between a visuospatial accuracy task and self‐reported confidence associated with decreased grey matter volume in the rostral anterior cingulate (Moeller *et al.* 2016). Deficits in self‐awareness of specific behaviours correlated with different brain regions; thus, impaired self‐awareness of apathy was associated with grey matter volume in the dorsal striatum, impulsivity with the orbitofrontal cortex and executive functioning with dorsolateral PFC in addiction (Moreno‐López *et al.* in press). Similar to our findings, impairments in self‐awareness improve with abstinence, including insight into the concordance between objective and subjective choices of drug‐related and non‐drug‐related cues (Moeller *et al.* 2010), self‐deception (Martínez‐González *et al.* 2016) and deficits in self‐awareness of frontal executive function (Verdejo‐García & Pérez‐García 2008).

Research suggests a partial overlap between the networks underlying intention awareness and goal‐directed behaviour, including lateral PFC and IPC (Lau *et al.* 2004; Sirigu *et al.* 2004; Gläscher *et al.* 2010) for goal‐directed behaviour and supplementary motor complex for habitual behaviours (Morris, Kundu, Dowell, *et al.* 2016b). The decrease in cognitive control implied by our data might be related to frontal anomalies observed in BD (Jacobus *et al.* 2009; McQueeny *et al.* 2009; Squeglia *et al.* 2012; Doallo *et al.* 2014; Vargas *et al.* 2014). Although partially reversible (Sullivan *et al.* 2005; Makris *et al.* 2008), the pernicious effects of alcohol on these structures are widely acknowledged. Both human and animal models have demonstrated that binge drinking compromises white matter fibre integrity in frontal and temporal regions (Jacobus *et al.* 2009; McQueeny *et al.* 2009; Vargas *et al.* 2014). Moreover, BD have been shown to have increased grey matter volumes in the VS (Howell *et al.* 2013), influenced by gender (Kvamme *et al.* 2016), and dlPFC (Doallo *et al.* 2014), and decreased cortical thickness in cingulate areas (Mashhoon *et al.* 2014). We have also recently shown that orientation dispersion index, a diffusion measure that putatively marks complexity of dendritic branching, in the same BD population under study demonstrates lower orientation dispersion index in regions implicated in higher‐order processing such as the dlPFC and parietal cortex and higher in the VS (Morris *et al.* in press). PFC input has been proven essential for both successful goal‐directed behaviour (Valentin *et al.* 2007; Tanaka *et al.* 2008; de Wit *et al.* 2009; Gläscher *et al.* 2010) and intention awareness (Lau *et al.* 2004), with model‐based learning further associated with efficient fronto‐striatal connectivity (de Wit *et al.* 2012; Morris, Kundu, Dowell, *et al.* 2016b).

From the perspective of action control, similar impairments in motor intention using the Libets clock task have been shown with Parkinson's disease, Tourette's syndrome and functional neurological disorders. These disorders are characterized by impaired self‐agency, or the sense of the loss of control over self‐generated actions. In particular, intriguing similarities may underlie the preceding sensory urge and associated motor tic in Tourette's syndrome and may provide insight into drug cue‐related urges triggered by sensory cue stimuli and subsequent motor (e.g. habitual or motivational) behaviour. The premonitory urge in Tourette's syndrome is involuntary and is commonly experienced as an involuntary sensory phenomenon (Cavanna *et al.* in press), and the tic itself has features of a voluntary movement in that its timing and decision to perform the tic movement in response to the urge can be controlled. A proportion of tics are preceded by a movement potential, a brain potential seen preceding voluntary movement, suggesting that tics have mixed features of voluntary and involuntary movements. The urge in tic disorders is similarly associated with activity in the anterior insula and anterior cingulate along with the supplementary motor area and parietal cortex (Bohlhalter *et al.* 2006; Lerner *et al.* 2007). A study focusing on the temporal generation of the urge demonstrated that the urge was associated with cortical activity at 2 seconds before the tic (supplementary motor area, primary sensory cortex and parietal cortex) and engaging subcortical regions 1 second before the tic (anterior cingulate cortex, putamen, insula, amygdala, cerebellum and occipital cortex) (Neuner *et al.* 2014). Tourette's syndrome has also been linked with lower gamma‐aminobutyric acid concentration in the sensory motor cortex along with decreased cortical thickness over the sensorimotor, insular and anterior cingulate cortices linked to the strength of premonitory urges (Draganski *et al.* 2010; Puts *et al.* 2015; Draper *et al.* 2016). Tourette's syndrome is also associated with enhanced habit formation correlating with greater structural connectivity of the supplementary motor cortex and sensorimotor putamen (Delorme *et al.* 2016).

The current study is not without limitations. Further characterization of the BD population in terms of whether they were hungover and further studies on mechanisms that might link the two including attentional mechanisms would be indicated. How these findings might relate to the literature on impaired awareness in substance misuse and the brain regions implicated would be useful. A prospective study using an alcohol challenge may be indicated.

Binge drinking is characterized by longer periods of abstinence between episodes of alcohol consumption (Courtney & Polich 2009). In the current sample of BD, not only was poorer intention awareness associated with alcohol use severity, but also subjects whose last binge drinking episode took place less than 4 days before testing displayed more model‐free habitual and less model‐based goal‐directed behaviour than those whose last binge drinking episode was more distant. In alcohol‐dependent subjects, abstinence has been observed to at least partially reverse some of the effects of alcohol abuse, at both the neuroanatomical and cognitive levels (Petry 2001; Sullivan *et al.* 2005; Makris *et al.* 2008; Voon, Derbyshire, *et al.* 2015b). Moreover, these findings dovetail with prior studies that have shown improvements of model‐based behaviours with abstinence in alcohol‐dependent subjects (Voon, Derbyshire, *et al.* 2015b). It is particularly interesting that this shift takes place over such a short period of time in BD. However, prior neuroanatomical findings suggest that BD might differ not only from HV but also from alcohol‐dependent subjects, with these differences possibly representing early sequelae, the compensatory effects of repeated binge and withdrawal patterns or even trait effects (Howell *et al.* 2013). The relationships observed here between intention awareness and alcohol use severity, and between model‐basedness, model‐freeness and abstinence suggest that top‐down volitional control might be ameliorated with alcohol use cessation.

### Author Contributions

ND is responsible for the data analysis and interpretation, and drafting of the manuscript and figures; DS, KB and PB performed the data acquisition and drafting of the manuscript; and VV is responsible for the study conception and design, interpretation of the data and drafting of the manuscript. All authors have critically reviewed content and approved final version submitted for publication.

References

Baek
K
, 
Doñamayor
N
, 
Morris
LS
, 
Strelchuk
D
, 
Mitchell
S
, 
Mikheenko
Y
, 
Yeoh
SY
, 
Phillips
W
, 
Zandi
M
, 
Jenaway
A
, 
Walsh
C
, 
Voon
V
 (in press) Impaired awareness of motor intention in functional neurological disorder: implications for voluntary and functional movement. Psychol Med. https://doi.org/10.1017/s0033291717000071.10.1017/S0033291717000071PMC596445928183377

Bari
A
, 
Robbins
TW
 (2013) Inhibition and impulsivity: behavioral and neural basis of response control. Prog Neurobiol
108:44–79. https://doi.org/10.1016/j.pneurobio.2013.06.005.2385662810.1016/j.pneurobio.2013.06.005

Bauer
LO
, 
Ceballos
NA
 (2014) Neural and genetic correlates of binge drinking among college women. Biol Psychol
97:43–48. https://doi.org/10.1016/j.biopsycho.2014.01.005.2453044010.1016/j.biopsycho.2014.01.005PMC3974158

Bohlhalter
S
, 
Goldfine
A
, 
Matteson
S
, 
Garraux
G
, 
Hanakawa
T
, 
Kansaku
K
, 
Wurzman
R
, 
Hallett
M
 (2006) Neural correlates of tic generation in Tourette syndrome: an event‐related functional MRI study. Brain
129:2029–2037. https://doi.org/10.1093/brain/awl050.1652033010.1093/brain/awl050

Cavanna
AE
, 
Black
KJ
, 
Hallett
M
, 
Voon
V
 (in press) Neurobiology of the premonitory urge in Tourette syndrome: pathophysiology and treatment implications. Journal of Neuropsychiatry and Clinical Neurosciences. https://doi.org/10.1176/appi.neuropsych.16070141.10.1176/appi.neuropsych.16070141PMC540910728121259

Corbit
LH
, 
Nie
H
, 
Janak
PH
 (2012) Habitual alcohol seeking: time course and the contribution of subregions of the dorsal striatum. Biol Psychiatry
72:389–395. https://doi.org/10.1016/j.biopsych.2012.02.024.2244061710.1016/j.biopsych.2012.02.024PMC3674580

Courtney
KE
, 
Polich
J
 (2009) Binge drinking in young adults: data, definitions, and determinants. Psychol Bull
135:142–156. https://doi.org/10.1037/a0014414.1921005710.1037/a0014414PMC2748736

Crabbe
JC
, 
Harris
RA
, 
Koob
GF
 (2011) Preclinical studies of alcohol binge drinking. Ann N Y Acad Sci
1216:24–40. https://doi.org/10.1111/j.1749‐6632.2010.05895.x.2127200910.1111/j.1749-6632.2010.05895.xPMC3076900

Crego
A
, 
Rodriguez‐Holguín
S
, 
Parada
M
, 
Mota
N
, 
Corral
M
, 
Cadaveira
F
 (2010) Reduced anterior prefrontal cortex activation in young binge drinkers during a visual working memory task. Drug Alcohol Depend
109:45–56. https://doi.org/10.1016/j.drugalcdep.2009.11.020.2007998010.1016/j.drugalcdep.2009.11.020

Danquah
AN
, 
Farrell
MJ
, 
O'Boyle
DJ
 (2008) Biases in the subjective timing of perceptual events: Libet *et al*. (1983) revisited. Conscious Cogn
17:616–627. https://doi.org/10.1016/j.concog.2007.09.005.1798376810.1016/j.concog.2007.09.005

Daw
ND
, 
Gershman
SJ
, 
Seymour
B
, 
Dayan
P
, 
Dolan
RJ
 (2011) Model‐based influences on humans' choices and striatal prediction errors. Neuron
69:1204–1215. https://doi.org/10.1016/j.neuron.2011.02.027.2143556310.1016/j.neuron.2011.02.027PMC3077926

de Wit
S
, 
Barker
RA
, 
Dickinson
AD
, 
Cools
R
 (2011) Habitual versus goal‐directed action control in Parkinson disease. J Cogn Neurosci
23:1218–1229. https://doi.org/10.1162/jocn.2010.21514.2042985910.1162/jocn.2010.21514

de Wit
S
, 
Corlett
PR
, 
Aitken
MRF
, 
Dickinson
A
, 
Fletcher
PC
 (2009) Differential engagement of the ventromedial prefrontal cortex by goal‐directed and habitual behavior toward food pictures in humans. J Neurosci
29:11330–11338. https://doi.org/10.1523/jneurosci.1639‐09.2009.1974113910.1523/JNEUROSCI.1639-09.2009PMC3443853

de Wit
S
, 
Watson
P
, 
Harsay
HA
, 
Cohen
MX
, 
van de Vijver
I
, 
Ridderinkhof
KR
 (2012) Corticostriatal connectivity underlies individual differences in the balance between habitual and goal‐directed action control. J Neurosci
32:12066–12075. https://doi.org/10.1523/jneurosci.1088‐12.2012.2293379010.1523/JNEUROSCI.1088-12.2012PMC6621537

Delorme
C
, 
Salvador
A
, 
Valabrègue
R
, 
Roze
E
, 
Palminteri
S
, 
Vidailhet
M
, 
de Wit
S
, 
Robbins
TW
, 
Hartmann
A
, 
Worbe
Y
 (2016) Enhanced habit formation in Gilles de la Tourette syndrome. Brain
139:605–615. https://doi.org/10.1093/brain/awv307.2649032910.1093/brain/awv307

Desmurget
M
, 
Sirigu
A
 (2012) Conscious motor intention emerges in the inferior parietal lobule. Curr Opin Neurobiol
22:1004–1011. https://doi.org/10.1016/j.conb.2012.06.006.2293956910.1016/j.conb.2012.06.006

Doallo
S
, 
Cadaveira
F
, 
Corral
M
, 
Mota
N
, 
López‐Caneda
E
, 
Holguín
SR
 (2014) Larger mid‐dorsolateral prefrontal gray matter volume in young binge drinkers revealed by voxel‐based morphometry. PLoS One
9:e96380. https://doi.org/10.1371/journal.pone.0096380.10.1371/journal.pone.0096380PMC400853224789323

Dolan
RJ
, 
Dayan
P
 (2013) Goals and habits in the brain. Neuron
80:312–325. https://doi.org/10.1016/j.neuron.2013.09.007.2413903610.1016/j.neuron.2013.09.007PMC3807793

Draganski
B
, 
Martino
D
, 
Cavanna
AE
, 
Hutton
C
, 
Orth
M
, 
Robertson
MM
, 
Critchley
HD
, 
Frackowiak
RS
 (2010) Multispectral brain morphometry in Tourette syndrome persisting into adulthood. Brain
133:3661–3675. https://doi.org/10.1093/brain/awq300.2107138710.1093/brain/awq300PMC2995885

Draper
A
, 
Jackson
GM
, 
Morgan
PS
, 
Jackson
SR
 (2016) Premonitory urges are associated with decreased grey matter thickness within the insula and sensorimotor cortex in young people with Tourette syndrome. J Neuropsychol
10:143–153. https://doi.org/10.1111/jnp.12089.2653828910.1111/jnp.12089PMC4982075

Edwards
MJ
, 
Moretto
G
, 
Schwingenschuh
P
, 
Katschnig
P
, 
Bhatia
KP
, 
Haggard
P
 (2011) Abnormal sense of intention preceding voluntary movement in patients with psychogenic tremor. Neuropsychologia
49:2791–2793. https://doi.org/10.1016/j.neuropsychologia.2011.05.021.2168372410.1016/j.neuropsychologia.2011.05.021

Everitt
BJ
, 
Robbins
TW
 (2013) From the ventral to the dorsal striatum: devolving views of their roles in drug addiction. Neuroscience & Biobehavioral Reviews
37:1946–1954. https://doi.org/10.1016/j.neubiorev.2013.02.010.2343889210.1016/j.neubiorev.2013.02.010

Fried
I
, 
Katz
A
, 
McCarthy
G
, 
Sass
KJ
, 
Williamson
P
, 
Spencer
SS
, 
Spencer
DD
 (1991) Functional organization of human supplementary motor cortex studied by electrical stimulation. J Neurosci
11:3656–3666.194110110.1523/JNEUROSCI.11-11-03656.1991PMC6575551

Fried
I
, 
Mukamel
R
, 
Kreiman
G
 (2011) Internally generated preactivation of single neurons in human medial frontal cortex predicts volition. Neuron
69:548–562. https://doi.org/10.1016/j.neuron.2010.11.045.2131526410.1016/j.neuron.2010.11.045PMC3052770

Gillan
CM
, 
Morein‐Zamir
S
, 
Urcelay
GP
, 
Sule
A
, 
Voon
V
, 
Apergis‐Schoute
AM
, 
Fineberg
NA
, 
Sahakian
BJ
, 
Robbins
TW
 (2014) Enhanced avoidance habits in obsessive–compulsive disorder. Biol Psychiatry
75:631–638. https://doi.org/10.1016/j.biopsych.2013.02.002.2351058010.1016/j.biopsych.2013.02.002PMC3988923

Gillan
CM
, 
Papmeyer
M
, 
Morein‐Zamir
S
, 
Sahakian
BJ
, 
Fineberg
NA
, 
Robbins
TW
, 
de Wit
S
 (2011) Disruption in the balance between goal‐directed behavior and habit learning in obsessive–compulsive disorder. Am J Psychiatry
168:718–726. https://doi.org/10.1176/appi.ajp.2011.10071062.2157216510.1176/appi.ajp.2011.10071062PMC3533260

Gläscher
J
, 
Daw
N
, 
Dayan
P
, 
O'Doherty
JP
 (2010) States versus rewards: dissociable neural prediction error signals underlying model‐based and model‐free reinforcement learning. Neuron
66:585–595. https://doi.org/10.1016/j.neuron.2010.04.016.2051086210.1016/j.neuron.2010.04.016PMC2895323

Gomes
G
 (1998) The timing of conscious experience: a critical review and reinterpretation of Libet's research. Conscious Cogn
7:559–595. https://doi.org/10.1006/ccog.1998.0332.981781410.1006/ccog.1998.0332

Hadj‐Bouziane
F
, 
Benatru
I
, 
Brovelli
A
, 
Klinger
H
, 
Thobois
S
, 
Broussolle
E
, 
Boussaoud
D
, 
Meunier
M
 (2013) Advanced Parkinson's disease effect on goal‐directed and habitual processes involved in visuomotor associative learning. Front Hum Neurosci
6
https://doi.org/10.3389/fnhum.2012.00351.2338681510.3389/fnhum.2012.00351PMC3560419

Haggard
P
, 
Libet
B
 (2001) Conscious intention and brain activity. Journal of Consciousness Studies
8:47–64.

Hester
R
, 
Nestor
L
, 
Garavan
H
 (2009) Impaired error awareness and anterior cingulate cortex hypoactivity in chronic cannabis users. Neuropsychopharmacology
34:2450–2458. https://doi.org/10.1038/npp.2009.67.1955391710.1038/npp.2009.67PMC2743772

Hogarth
L
, 
Chase
HW
, 
Baess
K
 (2012) Impaired goal‐directed behavioural control in human impulsivity. Q J Exp Psychol
65:305–316. https://doi.org/10.1080/17470218.2010.518242.10.1080/17470218.2010.518242PMC347132221077008

Howell
NA
, 
Worbe
Y
, 
Lange
I
, 
Tait
R
, 
Irvine
M
, 
Banca
P
, 
Harrison
NA
, 
Bullmore
ET
, 
Hutchison
WD
, 
Voon
V
 (2013) Increased ventral striatal volume in college‐aged binge drinkers. PLoS One
8:e74164. https://doi.org/10.1371/journal.pone.0074164.10.1371/journal.pone.0074164PMC378547424086317

Jacobus
J
, 
McQueeny
T
, 
Bava
S
, 
Schweinsburg
BC
, 
Frank
LR
, 
Yang
TT
, 
Tapert
SF
 (2009) White matter integrity in adolescents with histories of marijuana use and binge drinking. Neurotoxicol Teratol
31:349–355. https://doi.org/10.1016/j.ntt.2009.07.006.1963173610.1016/j.ntt.2009.07.006PMC2762024

Kvamme
TL
, 
Schmidt
C
, 
Strelchuk
D
, 
Chang‐Webb
YC
, 
Baek
K
, 
Voon
V
 (2016) Sexually dimorphic brain volume interaction in college‐aged binge drinkers. NeuroImage: Clinical
10:310–317. https://doi.org/10.1016/j.nicl.2015.12.004.2690057110.1016/j.nicl.2015.12.004PMC4724035

Lau
HC
, 
Rogers
RD
, 
Haggard
P
, 
Passingham
RE
 (2004) Attention to intention. Science
303:1208–1210. https://doi.org/10.1126/science.1090973.1497632010.1126/science.1090973

Lerner
A
, 
Bagic
A
, 
Boudreau
EA
, 
Hanakawa
T
, 
Pagan
F
, 
Mari
Z
, 
Bara‐Jimenez
W
, 
Aksu
M
, 
Garraux
G
, 
Simmons
JM
, 
Sato
S
, 
Murphy
DL
, 
Hallett
M
 (2007) Neuroimaging of neuronal circuits involved in tic generation in patients with Tourette syndrome. Neurology
68:1979–1987. https://doi.org/10.1212/01.wnl.0000264417.18604.12.1754854710.1212/01.wnl.0000264417.18604.12

Libet
B
 (1999) Do we have free will?
Journal of Consciousness Studies
6:47–57.

Libet
B
, 
Gleason
CA
, 
Wright
EW
, 
Pearl
DK
 (1983) Time of conscious intention to act in relation to onset of cerebral activity (readiness‐potential). Brain
106:623–642. https://doi.org/10.1093/brain/106.3.623.664027310.1093/brain/106.3.623

Makris
N
, 
Oscar‐Berman
M
, 
Jaffin
SK
, 
Hodge
SM
, 
Kennedy
DN
, 
Caviness
VS
, 
Marinkovic
K
, 
Breiter
HC
, 
Gasic
GP
, 
Harris
GJ
 (2008) Decreased volume of the brain reward system in alcoholism. Biol Psychiatry
64:192–202. https://doi.org/10.1016/j.biopsych.2008.01.018.1837490010.1016/j.biopsych.2008.01.018PMC2572710

Marsh
R
, 
Alexander
GM
, 
Packard
MG
, 
Zhu
H
, 
Wingard
JC
, 
Quackenbush
G
, 
Peterson
BS
 (2004) Habit learning in Tourette syndrome: a translational neuroscience approach to a developmental psychopathology. Arch Gen Psychiatry
61:1259–1268. https://doi.org/10.1001/archpsyc.61.12.1259.1558311710.1001/archpsyc.61.12.1259

Martínez‐González
JM
, 
Vilar López
R
, 
Becoña Iglesias
E
, 
Verdejo‐García
A
 (2016) Self‐deception as a mechanism for the maintenance of drug addiction. Psicothema
28:13–19. https://doi.org/10.7334/psicothema2015.139.2682041810.7334/psicothema2015.139

Mashhoon
Y
, 
Czerkawski
C
, 
Crowley
DJ
, 
Cohen‐Gilbert
JE
, 
Sneider
JT
, 
Silveri
MM
 (2014) Binge alcohol consumption in emerging adults: anterior cingulate cortical “thinness” is associated with alcohol use patterns. Alcohol Clin Exp Res
38:1955–1964. https://doi.org/10.1111/acer.12475.2496187110.1111/acer.12475PMC4107054

McQueeny
T
, 
Schweinsburg
BC
, 
Schweinsburg
AD
, 
Jacobus
J
, 
Bava
S
, 
Frank
LR
, 
Tapert
SF
 (2009) Altered white matter integrity in adolescent binge drinkers. Alcohol Clin Exp Res
33:1278–1285. https://doi.org/10.1111/j.1530-0277.2009.00953.x.1938918510.1111/j.1530-0277.2009.00953.xPMC2825379

Moeller
SJ
, 
Fleming
SM
, 
Gan
G
, 
Zilverstand
A
, 
Malaker
P
, 
d'Oleire Uquillas
F
, 
Schneider
KE
, 
Preston‐Campbell
RN
, 
Parvaz
MA
, 
Maloney
T
, 
Alia‐Klein
N
, 
Goldstein
RZ
 (2016) Metacognitive impairment in active cocaine use disorder is associated with individual differences in brain structure. Eur Neuropsychopharmacol
26:653–662. https://doi.org/10.1016/j.euroneuro.2016.02.009.2694866910.1016/j.euroneuro.2016.02.009PMC4805109

Moeller
SJ
, 
Maloney
T
, 
Parvaz
MA
, 
Alia‐Klein
N
, 
Woicik
PA
, 
Telang
F
, 
Wang
GJ
, 
Volkow
ND
, 
Goldstein
RZ
 (2010) Impaired insight in cocaine addiction: laboratory evidence and effects on cocaine‐seeking behaviour. Brain
133:1484–1493. https://doi.org/10.1093/brain/awq066.2039526410.1093/brain/awq066PMC2912695

Moreno‐López
L
, 
Albein‐Urios
N
, 
Martínez‐González
JM
, 
Soriano‐Mas
C
, 
Verdejo‐García
A
 (in press) Neural correlates of impaired self‐awareness of apathy, disinhibition and dysexecutive deficits in cocaine‐dependent individuals. Addict Biol. https://doi.org/10.1111/adb.12422.10.1111/adb.1242227397847

Moretto
G
, 
Schwingenschuh
P
, 
Katschnig
P
, 
Bhatia
KP
, 
Haggard
P
 (2011) Delayed experience of volition in Gilles de la Tourette syndrome. J Neurol Neurosurg Psychiatry. https://doi.org/10.1136/jnnp.2010.221143.10.1136/jnnp.2010.22114321212106

Morris
LS
, 
Dowell
NG
, 
Cercignani
M
, 
Harrison
NA
, 
Voon
V
 (in press) Binge drinking differentially affects cortical and subcortical microstructure. Addiction Biology. https://doi.org/10.1111/adb.12493.10.1111/adb.12493PMC581182128105707

Morris
LS
, 
Kundu
P
, 
Baek
K
, 
Irvine
MA
, 
Mechelmans
DJ
, 
Wood
J
, 
Harrison
NA
, 
Robbins
TW
, 
Bullmore
ET
, 
Voon
V
 (2016a) Jumping the gun: mapping neural correlates of waiting impulsivity and relevance across alcohol misuse. Biol Psychiatry
79:499–507. https://doi.org/10.1016/j.biopsych.2015.06.009.2618501010.1016/j.biopsych.2015.06.009PMC4764648

Morris
LS
, 
Kundu
P
, 
Dowell
N
, 
Mechelmans
DJ
, 
Favre
P
, 
Irvine
MA
, 
Robbins
TW
, 
Daw
N
, 
Bullmore
ET
, 
Harrison
NA
, 
Voon
V
 (2016b) Fronto‐striatal organization: defining functional and microstructural substrates of behavioural flexibility. Cortex
74:118–133. https://doi.org/10.1016/j.cortex.2015.11.004.2667394510.1016/j.cortex.2015.11.004PMC4729321

Naqvi
NH
, 
Rudrauf
D
, 
Damasio
H
, 
Bechara
A
 (2007) Damage to the insula disrupts addiction to cigarette smoking. Science
315:531–534. https://doi.org/10.1126/science.1135926.1725551510.1126/science.1135926PMC3698854
National Institute on Alcohol Abuse and Alcoholism
. (2004, Winter). NIAAA council approves definition of binge drinking
*NIAAA Newsletter* (Vol. 3, pp. 3).

Neuner
I
, 
Werner
CJ
, 
Arrubla
J
, 
Stöcker
T
, 
Ehlen
C
, 
Wegener
HP
, 
Schneider
F
, 
Shah
NJ
 (2014) Imaging the where and when of tic generation and resting state networks in adult Tourette patients. Front Hum Neurosci
8
https://doi.org/10.3389/fnhum.2014.00362.10.3389/fnhum.2014.00362PMC403575624904391

Okita
K
, 
Ghahremani
DG
, 
Payer
DE
, 
Robertson
CL
, 
Mandelkern
MA
, 
London
ED
 (2016) Relationship of alexithymia ratings to dopamine D_2_‐type receptors in anterior cingulate and insula of healthy control subjects but not methamphetamine‐dependent individuals. International Journal of Neuropsychopharmacology
19
https://doi.org/10.1093/ijnp/pyv129.pyv129.10.1093/ijnp/pyv129PMC488666826657175

Paulus
MP
, 
Stewart
JL
 (2014) Interoception and drug addiction. Neuropharmacology
76:342–350. https://doi.org/10.1016/j.neuropharm.2013.07.002.2385599910.1016/j.neuropharm.2013.07.002PMC3858461

Petry
NM
 (2001) Delay discounting of money and alcohol in actively using alcoholics, currently abstinent alcoholics, and controls. Psychopharmacology (Berl)
154:243–250. https://doi.org/10.1007/s002130000638.1135193110.1007/s002130000638

Puts
NAJ
, 
Harris
AD
, 
Crocetti
D
, 
Nettles
C
, 
Singer
HS
, 
Tommerdahl
M
, 
Edden
RAE
, 
Mostofsky
SH
 (2015) Reduced GABAergic inhibition and abnormal sensory symptoms in children with Tourette syndrome. J Neurophysiol
114:808
https://doi.org/10.1152/jn.00060.2015.2604182210.1152/jn.00060.2015PMC4533064

Sanchez‐Roige
S
, 
Baro
V
, 
Trick
L
, 
Pena‐Oliver
Y
, 
Stephens
DN
, 
Duka
T
 (2014) Exaggerated waiting impulsivity associated with human binge drinking, and high alcohol consumption in mice. Neuropsychopharmacology
39:2919–2927. https://doi.org/10.1038/npp.2014.151.2494790110.1038/npp.2014.151PMC4229569

Sebold
M
, 
Deserno
L
, 
Nebe
S
, 
Schad
DJ
, 
Garbusow
M
, 
Hägele
C
, 
Keller
J
, 
Jänger
E
, 
Kathmann
N
, 
Smolka
MN
, 
Rapp
MA
, 
Schlagenhauf
F
, 
Heinz
A
, 
Huys
QJM
 (2014) Model‐based and model‐free decisions in alcohol dependence. Neuropsychobiology
70:122–131. https://doi.org/10.1159/000362840.2535949210.1159/000362840

Senatorov
VV
, 
Damadzic
R
, 
Mann
CL
, 
Schwandt
ML
, 
George
DT
, 
Hommer
DW
, 
Heilig
M
, 
Momenan
R
 (2015) Reduced anterior insula, enlarged amygdala in alcoholism and associated depleted von Economo neurons. Brain
138:69–79. https://doi.org/10.1093/brain/awu305.2536702210.1093/brain/awu305PMC4285187

Sheehan
DV
, 
Lecrubier
Y
, 
Sheehan
KH
, 
Amorim
P
, 
Janavs
J
, 
Weiller
E
, 
Hergueta
T
, 
Baker
R
, 
Dunbar
GC
 (1998) The mini‐international neuropsychiatric interview (M.I.N.I): the development and validation of a structured diagnostic psychiatric interview for DSM‐IV and ICD‐10. J Clin Psychiatry
59:22–33.9881538

Sirigu
A
, 
Daprati
E
, 
Ciancia
S
, 
Giraux
P
, 
Nighoghossian
N
, 
Posada
A
, 
Haggard
P
 (2004) Altered awareness of voluntary action after damage to the parietal cortex. Nat Neurosci
7:80–84. https://doi.org/10.1038/nn1160.1464729010.1038/nn1160

Sjoerds
Z
, 
de Wit
S
, 
van den Brink
W
, 
Robbins
TW
, 
Beekman
ATF
, 
Penninx
BWJH
, 
Veltman
DJ
 (2013) Behavioral and neuroimaging evidence for overreliance on habit learning in alcohol‐dependent patients. Transl Psychiatry
3:e337. https://doi.org/10.1038/tp.2013.107.10.1038/tp.2013.107PMC403032624346135

Squeglia
L
, 
Sorg
S
, 
Schweinsburg
A
, 
Wetherill
R
, 
Pulido
C
, 
Tapert
S
 (2012) Binge drinking differentially affects adolescent male and female brain morphometry. Psychopharmacology (Berl)
220:529–539. https://doi.org/10.1007/s00213‐011‐2500‐4.2195266910.1007/s00213-011-2500-4PMC3527131

Stewart
JL
, 
May
AC
, 
Poppa
T
, 
Davenport
PW
, 
Tapert
SF
, 
Paulus
MP
 (2014) You are the danger: attenuated insula response in methamphetamine users during aversive interoceptive decision‐making. Drug & Alcohol Dependence
142:110–119. https://doi.org/10.1016/j.drugalcdep.2014.06.003.2499318610.1016/j.drugalcdep.2014.06.003PMC4127120

Sullivan
EV
, 
Deshmukh
A
, 
De Rosa
E
, 
Rosenbloom
MJ
, 
Pfefferbaum
A
 (2005) Striatal and forebrain nuclei volumes: contribution to motor function and working memory deficits in alcoholism. Biol Psychiatry
57:768–776. https://doi.org/10.1016/j.biopsych.2004.12.012.1582023410.1016/j.biopsych.2004.12.012

Tabu
H
, 
Aso
T
, 
Matsuhashi
M
, 
Ueki
Y
, 
Takahashi
R
, 
Fukuyama
H
, 
Shibasaki
H
, 
Mima
T
 (2015) Parkinson's disease patients showed delayed awareness of motor intention. Neurosci Res
95:74–77. https://doi.org/10.1016/j.neures.2015.01.012.2564666710.1016/j.neures.2015.01.012

Tanaka
SC
, 
Balleine
BW
, 
O'Doherty
JP
 (2008) Calculating consequences: brain systems that encode the causal effects of actions. J Neurosci
28:6750–6755. https://doi.org/10.1523/jneurosci.1808‐08.2008.1857974910.1523/JNEUROSCI.1808-08.2008PMC3071565

Valentin
VV
, 
Dickinson
A
, 
O'Doherty
JP
 (2007) Determining the neural substrates of goal‐directed learning in the human brain. J Neurosci
27:4019–4026. https://doi.org/10.1523/jneurosci.0564‐07.2007.1742897910.1523/JNEUROSCI.0564-07.2007PMC6672546

van der Meer
MAA
, 
Redish
AD
 (2010) Expectancies in decision making, reinforcement learning, and ventral striatum. Front Neurosci
3
https://doi.org/10.3389/neuro.01.006.2010.10.3389/neuro.01.006.2010PMC289148521221409

Vargas
WM
, 
Bengston
L
, 
Gilpin
NW
, 
Whitcomb
BW
, 
Richardson
HN
 (2014) Alcohol binge drinking during adolescence or dependence during adulthood reduces prefrontal myelin in male rats. J Neurosci
34:14777–14782. https://doi.org/10.1523/jneurosci.3189‐13.2014.2535522910.1523/JNEUROSCI.3189-13.2014PMC4212071

Verdejo‐García
A
, 
Clark
L
, 
Dunn
BD
 (2012) The role of interoception in addiction: a critical review. Neuroscience & Biobehavioral Reviews
36:1857–1869. https://doi.org/10.1016/j.neubiorev.2012.05.007.2265964210.1016/j.neubiorev.2012.05.007

Verdejo‐García
A
, 
Pérez‐García
M
 (2008) Substance abusers' self‐awareness of the neurobehavioral consequences of addiction. Psychiatry Res
158:172–180. https://doi.org/10.1016/j.psychres.2006.08.001.1823778610.1016/j.psychres.2006.08.001

Vollstädt‐Klein
S
, 
Wichert
S
, 
Rabinstein
J
, 
Bühler
M
, 
Klein
O
, 
Ende
G
, 
Hermann
D
, 
Mann
K
 (2010) Initial, habitual and compulsive alcohol use is characterized by a shift of cue processing from ventral to dorsal striatum. Addiction
105:1741–1749. https://doi.org/10.1111/j.1360‐0443.2010.03022.x.2067034810.1111/j.1360-0443.2010.03022.x

Voon
V
, 
Baek
K
, 
Enander
J
, 
Worbe
Y
, 
Morris
LS
, 
Harrison
NA
, 
Robbins
TW
, 
Rück
C
, 
Daw
ND
 (2015a) Motivation and value influences in the relative balance of goal‐directed and habitual behaviours in obsessive–compulsive disorder. Transl Psychiatry
5:e670. https://doi.org/10.1038/tp.2015.165.10.1038/tp.2015.165PMC506875826529423

Voon
V
, 
Derbyshire
K
, 
Rück
C
, 
Irvine
MA
, 
Worbe
Y
, 
Enander
J
, 
Schreiber
LRN
, 
Gillan
CM
, 
Fineberg
NA
, 
Sahakian
BJ
, 
Robbins
TW
, 
Harrison
NA
, 
Wood
J
, 
Daw
ND
, 
Dayan
P
, 
Grant
JE
, 
Bullmore
ET
 (2015b) Disorders of compulsivity: a common bias towards learning habits. Mol Psychiatry
20:345–352. https://doi.org/10.1038/mp.2014.44.2484070910.1038/mp.2014.44PMC4351889

Walsh
E
, 
Kühn
S
, 
Brass
M
, 
Wenke
D
, 
Haggard
P
 (2010) EEG activations during intentional inhibition of voluntary action: an electrophysiological correlate of self‐control?
Neuropsychologia
48:619–626. https://doi.org/10.1016/j.neuropsychologia.2009.10.026.1988366710.1016/j.neuropsychologia.2009.10.026

Whelan
R
, 
Watts
R
, 
Orr
CA
, 
Althoff
RR
, 
Artiges
E
, 
Banaschewski
T
, 
Barker
GJ
, 
Bokde
ALW
, 
Büchel
C
, 
Carvalho
FM
, 
Conrod
PJ
, 
Flor
H
, 
Fauth‐Buhler
M
, 
Frouin
V
, 
Gallinat
J
, 
Gan
G
, 
Gowland
P
, 
Heinz
A
, 
Ittermann
B
, 
Lawrence
C
, 
Mann
K
, 
Martinot
JL
, 
Nees
F
, 
Ortiz
N
, 
Paillere‐Martinot
ML
, 
Paus
T
, 
Pausova
Z
, 
Rietschel
M
, 
Robbins
TW
, 
Smolka
MN
, 
Strohle
A
, 
Schumann
G
, 
Garavan
H
, 
the Imagen Consortium
(2014) Neuropsychosocial profiles of current and future adolescent alcohol misusers. Nature
512:185–189. https://doi.org/10.1038/nature13402.2504304110.1038/nature13402PMC4486207

Worbe
Y
, 
Irvine
M
, 
Lange
I
, 
Kundu
P
, 
Howell
NA
, 
Harrison
NA
, 
Bullmore
ET
, 
Robbins
TW
, 
Voon
V
 (2014) Neuronal correlates of risk‐seeking attitudes to anticipated losses in binge drinkers. Biol Psychiatry
76:717–724. https://doi.org/10.1016/j.biopsych.2013.11.028.2438782210.1016/j.biopsych.2013.11.028PMC4192134

Wylie
SA
, 
Claassen
DO
, 
Kanoff
KE
, 
Ridderinkhof
KR
, 
van den Wildenberg
WPM
 (2013) Impaired inhibition of prepotent motor actions in patients with Tourette syndrome. Journal of Psychiatry and Neuroscience
38:349–356. https://doi.org/10.1503/jpn.120138.2382018510.1503/jpn.120138PMC3756119
